# T2Candida Assay in the Diagnosis of Intraabdominal Candidiasis: A Prospective Multicenter Study

**DOI:** 10.3390/jof8010086

**Published:** 2022-01-16

**Authors:** Anders Krifors, Måns Ullberg, Markus Castegren, Johan Petersson, Ernesto Sparrelid, Helena Hammarström, Jan Sjölin, Volkan Özenci, Ola Blennow

**Affiliations:** 1 Department of Physiology and Pharmacology, Karolinska Institutet, 171 65 Stockholm, Sweden; markus.castegren@medsci.uu.se (M.C.); johan.petersson@regionstockholm.se (J.P.); 2 Centre for Clinical Research Västmanland, Uppsala University, Hospital of Västmanland, 721 89 Västerås, Sweden; 3 Department of Clinical Microbiology, Karolinska University Hospital, 171 76 Stockholm, Sweden; mans.ullberg@regionstockholm.se (M.U.); volkan.ozenci@regionstockholm.se (V.Ö.); 4 Perioperative Medicine and Intensive Care (PMI), Karolinska University Hospital, 171 76 Stockholm, Sweden; 5 Department of Clinical Science, Intervention and Technology (CLINTEC), Karolinska Institutet, Karolinska University Hospital, 141 52 Stockholm, Sweden; ernesto.sparrelid@regionstockholm.se; 6 Department of Infectious Diseases, Institute of Biomedicine, Sahlgrenska Academy, University of Gothenburg, 402 34 Gothenburg, Sweden; helena.hammarstrom@vgregion.se; 7 Department of Infectious Diseases, Region Västra Götaland, Sahlgrenska University Hospital, 402 34 Gothenburg, Sweden; 8 Department of Medical Sciences, Section of Infectious Diseases, Uppsala University, 751 85 Uppsala, Sweden; jan.sjolin@gmail.com; 9 Department of Laboratory Medicine, Division of Clinical Microbiology, Karolinska Institutet, 141 52 Stockholm, Sweden; 10 Department of Infectious Diseases, Karolinska University Hospital, 171 76 Stockholm, Sweden; ola.blennow@regionstockholm.se

**Keywords:** T2 magnetic resonance, beta-glucan, blood cultures, intraabdominal candidiasis, invasive candidiasis

## Abstract

The T2Candida magnetic resonance assay is a direct-from-blood pathogen detection assay that delivers a result within 3–5 h, targeting the most clinically relevant *Candida* species. Between February 2019 and March 2021, the study included consecutive patients aged >18 years admitted to an intensive care unit or surgical high-dependency unit due to gastrointestinal surgery or necrotizing pancreatitis and from whom diagnostic blood cultures were obtained. Blood samples were tested in parallel with T2Candida and 1,3-β-D-glucan. Of 134 evaluable patients, 13 (10%) were classified as having proven intraabdominal candidiasis (IAC) according to the EORTC/MSG criteria. Two of the thirteen patients (15%) had concurrent candidemia. The sensitivity, specificity, positive predictive value, and negative predictive value, respectively, were 46%, 97%, 61%, and 94% for T2Candida and 85%, 83%, 36%, and 98% for 1,3-β-D-glucan. All positive T2Candida results were consistent with the culture results at the species level, except for one case of dual infection. The performance of T2Candida was comparable with that of 1,3-β-D-glucan for candidemic IAC but had a lower sensitivity for non-candidemic IAC (36% vs. 82%). In conclusion, T2Candida may be a valuable complement to 1,3-β-D-glucan in the clinical management of high-risk surgical patients because of its rapid results and ease of use.

## 1. Introduction

Invasive candidiasis (IC) is a severe infection associated with an attributable mortality of up to 40% [1,2]. The incidence of candidemia is increasing among patients in the intensive care unit (ICU), accounting for approximately 5–15% of all bloodstream infections [3]. Even though effective antifungal therapy is available, a successful outcome depends on a timely diagnosis, prompt initiation of an appropriate treatment, and source control [4]. In the ICU, IC is generally observed as candidemia or intraabdominal candidiasis (IAC) related to intraabdominal surgery or necrotizing pancreatitis [5,6,7]. Diagnosing IC can be challenging as traditional diagnostic cultures have been reported to have a low diagnostic yield, as low as around 50% in blood cultures, and a slow turnaround time of 2–4 days [8]. Empiric antifungal therapy may lead to costly overtreatment with potentially harmful drugs as well as antifungal resistance. Therefore, efforts have been made to facilitate the early diagnosis of IC using screening protocols and novel diagnostic methods [9]. The detection of 1,3-β-D-glucan (BDG), a fungal cell wall component, may support IC diagnosis but must be interpreted in a proper clinical context since BDG is not *Candida*-specific and false-positive results due to concomitant therapies or infections have been reported [10,11].

The T2Candida magnetic resonance assay (T2C) uses a novel technique to detect *Candida* cells directly in whole blood [12]. T2C lyses the *Candida* cells by mechanical bead beating, amplifies Candida DNA using a thermostable DNA polymerase, and clusters the DNA to magnetic nanoparticles that can be detected due to the changes in the magnetic resonance. The five most clinically relevant *Candida* species are targeted, and they can be detected at a density of only 1 CFU/mL with a turnaround-time of only 3–5 h [13]. In contrast to BDG, T2C can provide information at the species level, thereby helping in choosing the treatment. A pooled analysis of eight studies reported T2C sensitivity and specificity for candidemia of 91% and 94%, respectively, but it did not include non-candidemic IAC [14].

There is a lack of prospective clinical studies evaluating the performance of the T2C assay in surgical patients with IAC. The primary aim of the present study is to prospectively evaluate the diagnostic performance of the T2C assay in patients with proven or probable IAC. A secondary aim is to compare the performance of T2C with that of the BDG assay in patients with proven IAC.

## 2. Materials and Methods

This prospective observational multicenter study was conducted at the ICU and surgical high dependency unit (HDU) of the Karolinska University Hospitals, Huddinge and Solna, Stockholm; at the ICU at Sahlgrenska University Hospitals, Östra and Sahlgrenska, Gothenburg; and at the ICU at Västerås Hospital, Västerås. Between February 2019 and March 2021, consecutive patients aged >18 years who were admitted to the ICU/HDU after gastrointestinal surgery or due to necrotizing pancreatitis and who had diagnostic blood cultures obtained because of suspicion of infection were tested in parallel using both T2C and BDG. The selected study population was determined based on the assumption that the pre-test probability of IAC would exceed 10%. The results of all of the tests were made clinically available. In addition, TC2 and BDG re-testing was performed each time new blood cultures were collected.

Blood cultures were analyzed at the microbiology department in each study center using BacT/ALERT FA Plus Aerobic and FN Plus Anaerobic (bioMérieux, Marcy l’Etoile, France) and incubated in the BacT/ALERT VIRTUO Culture System (bioMérieux, Marcy l’Etoile, France) for a maximum period of 7 to 10 days depending on the center. Positive blood cultures were subjected to gram stain microscopy, sub-culturing, and analysis using direct-matrix assisted laser desorption/ionization–time-of-flight mass spectrometry (MALDI–TOF MS) and subsequent MALDI–TOF from single spotted colonies.

T2C testing was performed using 3 mL of whole blood collected in an EDTA container and inserted into the fully automatic T2Dx instrument (T2Biosystems, Lexington, KY, USA) according to the manufacturer’s instructions. Positive results were reported as follows: *C. albicans*/*C. tropicalis*, *C. glabrata*/*C. krusei*, and *C. parapsilosis*. Failure of the internal control resulted in an “invalid” result. BDG testing was performed using the Fungitell assay (Cape Cod, East Falmouth, MA, USA) according to the manufacturer’s instructions, with a positivity cut-off ≥ 100 pg/mL.

Clinical data, supportive microbiological evidence, and outcome measures were retrospectively collected from the patient medical records using a case report form.

The study was approved by the Swedish Ethical Review Board (DNR 2019-00603).

### Definition of Proven and Probable IAC

Proven IAC was defined according to the recently updated EORTC/MSG criteria, requiring growth of *Candida* spp. in a sample obtained by a sterile procedure from a normally sterile site, including a sample from a drain inserted within 24 h and showing clinical or radiological abnormality consistent with an infectious process (Table 1) [15]. Only samples obtained within ±7 days from testing were considered for the case classification. According to the EORTC/MSG criteria, the definition of probable IC is only applicable in immunocompromised patients. However, in the accompanying text, consecutive positive BDG tests using a threshold value of ≥ 80 pg/mL were considered suitable to define probable IC in a high-risk ICU population with a prevalence of more than 10% [15]. An initiative to define suitable criteria for IC specifically in an ICU population has recently been published [16]. Proposed host criteria included patients with abdominal surgery or necrotizing pancreatitis, with one of the proposed mycological criteria for probable IC being consecutive positive BDG tests. In the present study, these definitions for probable IAC were used with the only difference being that the mycological criteria were defined as fulfilled by a single positive BDG test using a threshold value of ≥ 100 pg/mL (Table 1). To improve the specificity of the classification, patients with probable IAC were also subcategorized according to treatment with antifungals or the presence of supportive cultures growing *Candida* spp. within ±7 days. Patients fulfilling neither proven IAC criteria nor the criteria for having at least one BDG test ≥ 100 pg/mL were classified as negative IAC.

## 3. Results

**Figure 1:**
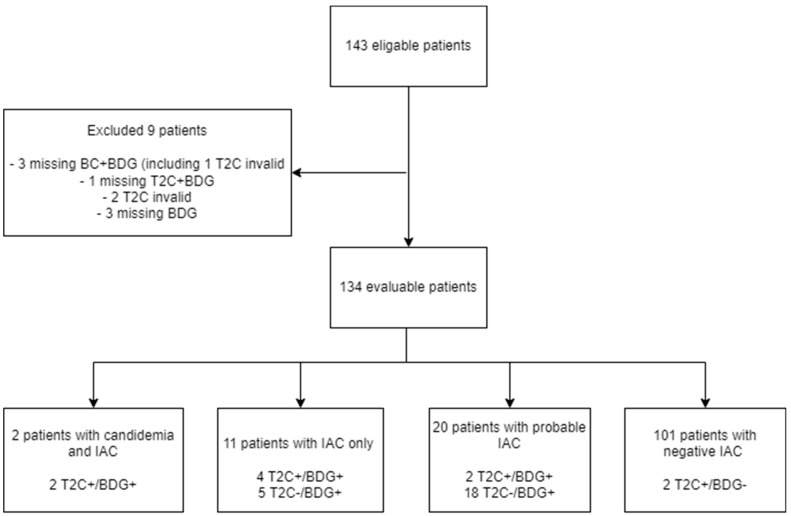
Flowchart of patient inclusion. Abbreviations: T2C, T2Candida magnetic resonance assay; BDG, 1,3-β-D-glucan; IAC, intraabdominal *candidiasis*.

Of the 143 consecutive patients intended for inclusion, 9 patients were excluded due to missing one or more of the diagnostic tests (blood culture, T2C or BDG; *n* = 7) and/or an invalid T2C result (*n* = 3), leaving 134 evaluable patients (Figure 1). The median age was 66 years, 57% were men, and the majority (87%) had preceding gastrointestinal surgery (Table 2).

### 3.1. Performance of T2C and BDG in Proven IAC

Of the 134 patients (10%), 13 patients fulfilled the definitions for proven IAC (Table 3). Two patients had proven IAC and concomitant candidemia, and eleven had proven IAC only. The *Candida* species isolated in the patients with proven IAC were *C. albicans* (*n* = 8), including two patients with concomitant candidemia; *C. parapsilosis* (*n* = 2); *C. tropicalis* (*n* = 1); *C. glabrata* (*n* = 1); and one dual infection with *C. albicans* and *C. parapsilosis*. Of these 13 (46%) patients, 6 patients were receiving antifungal therapy at the time of testing.

T2C and BDG were positive in the two patients with IAC and concomitant candidemia. In non-candidemic IAC, T2C was positive in 4/11 (36%) patients compared with 9/11 (82%) for BDG (Figure 1 and Table 3). All T2C positive patients also tested BDG positive. T2C correctly identified *Candida* on a species level in all patients except for one non-candidemic IAC patient with growth of both *C. albicans* and *C. parapsilosis*, where T2C identified the presence of only *C. albicans*/*C. tropicalis*. The average BDG value among proven IC cases was 1568 pg/mL with a median of 490 pg/mL (range < 31 pg/mL to 14,470 pg/mL).

Overall, 8/13 patients with proven IAC were admitted to the ICU at the time of testing (average Sequential Organ Failure Assessment (SOFA) score of 5.0), with 5/8 patients being T2C positive compared with 1/5 patients with proven IAC admitted to the HDU being T2C positive (average SOFA score of 1.6). Table 4 shows that the sensitivity and the specificity for proven IAC were 46% and 97% for T2C and 85% and 83% for BDG, respectively. Positive and negative predictive values (PPV and NPV) with an estimated disease prevalence of 1, 5, and 10% are presented in Table 4.

### 3.2. Performance of T2C in Probable IAC

Twenty patients were classified as having probable IAC based on a single BDG value of ≥ 100 pg/mL, with an average BDG of 1145 pg/mL and a median of 364 pg/mL (range 115 pg/mL to 5000 pg/mL). Eleven (55%) of these patients were receiving antifungal therapy at the time of testing or within 7 days afterward. Ten (50%) patients had at least one culture with the growth of *Candida* spp. within ±7 days from inclusion, either in a drain culture obtained >24 h from insertion or a culture from a non-sterile location, thus not meeting the criteria for proven infection.

Two patients were T2C positive; both received antifungal therapy and had at least one positive supportive culture.

Sensitivity, specificity, PPV, and NPV for proven and probable IAC are presented in Table 4 with the results of the more confined definitions of probable IAC (positive BDG in combination with antifungal treatment and positive BDG in combination with supportive culture not fulfilling proven criteria).

### 3.3. T2C in Serial Testing

In total, 24 patients were re-tested, with 16/24 (67%) being tested within 7 days. No patients who were T2C negative in the first test became positive in the second test. Of the 10 T2C positive patients, 6 patients were re-tested within 7 days and 4 remained T2C positive. Three of these patients fulfilled proven IAC criteria at the time of first testing, while the fourth patient, who was classified as IAC negative when tested two days earlier, developed candidemia at the time of second testing. Two of three T2C-positive patients remained positive in T2C in a third consecutive test where serial blood cultures were negative, including one of the candidemia cases.

## 4. Discussion

In the present study, we included 134 consecutive high-risk surgical patients treated in ICU/HDU with suspicion of a new infection. It is the largest prospective study focusing on the performance of T2C in the diagnosis of IAC to date. The incidence of proven IAC was 10% and T2C detected both cases of IAC with concomitant candidemia but only 36% of non-candidemic IAC. BDG had a markedly higher sensitivity of 85%, although at the cost of lower specificity of 83%, compared with 97% for T2C. These results are in line with a recent ICU study from Switzerland that included 48 high-risk surgical patients with a reported sensitivity of 33% for T2C and of 83% for BDG in diagnosing IAC [17]. The lower sensitivity of T2C compared with BDG in diagnosing proven IAC may be due to deep-seated infections only intermittently seeding *Candida* cells into the bloodstream. In contrast, BDG may leak into the circulation and, because of its long half-life, remain there at a high concentration for longer periods [18].

Four out of six T2C-positive patients with proven IAC were receiving antifungal therapy at the time of testing. One IAC patient with concomitant candidemia remained T2C positive three consecutive times over 10 days despite ongoing antifungal therapy and negative follow-up blood cultures. T2C correctly identified the *Candida* spp. in all six cases, missing *C. parapsilosis* in one patient with a double infection with *C. albicans* and *C. parapsilosis*. These findings indicate that T2C may also be useful after empiric antifungal therapy has been initiated. This is consistent with previous reports that the T2C performance was maintained in the presence of antifungals [19,20].

Interestingly, T2C appeared to perform better in critically ill patients. Among patients admitted to ICU (median SOFA score 5), 63% of patients with proven IAC were T2C positive, compared with 20% of patients admitted to HDU (median SOFA score 1.6). One possible explanation may be that seeding of *Candida* cells into circulation occurs more often in critically ill patients with IAC, especially in patients with inadequate source control. Early effective antifungal therapy in combination with adequate source control is critical for a successful outcome in IAC [6,21].

Using only proven IAC when evaluating the performance of T2C and BDG may underestimate the true incidence of IAC as blood cultures have a sensitivity of only 5–20% for IAC, and it is not always possible to obtain deep cultures or tissue samples [22]. Thus, evaluating the performance of T2C for proven IAC in combination with probable IAC may be a more clinically relevant comparison. Even though no international consensus criteria exist for probable IAC in non-immunocompromised patients, leading experts have recently proposed such criteria and included high-risk surgery patients with consecutive positive BDG [16]. All patients in the present study fulfilled the proposed high-risk surgery criteria, but a modification of the mycological criteria to one positive BDG test with a ≥100 pg/mL threshold was required since only one BDG result was available in most cases. By this definition, the sensitivity for T2C was lowered from 46% for proven IAC to 24% for the combination of proven and probable IAC. Subcategorizing probable IAC further by only including whether the patient had received antifungal therapy or had a positive supportive culture within seven days of testing, led to a modest increase in sensitivity, but it was still low at 32% and 35%, respectively. The implication of these findings appear to be that the value of T2C in diagnosing IAC in patient groups other than severely ill high-risk surgical patients in ICU is limited. Comparing the performances of T2C and BDG in this study, it appears that BDG with higher sensitivity but lower specificity and T2C with lower sensitivity but higher specificity complement each other in diagnosing IAC. This is consistent with the results in the above-mentioned study by Lamoth et al. [17], who found that, in 48 high-risk surgical patients, IAC was present in 100% of patients with positive BDG/T2C and absent in 90% of patients with negative BDG/T2C.

Our study has several limitations. First, there were only 13/134 (10%) proven IAC which makes comparative statistical inference difficult. However, the included cohort met the anticipated prevalence of 10% IAC, and even though the study represents the largest study to date, a significantly larger study would be required for this purpose. Second, we used a BDG positivity cut-off value of ≥100 pg/mL instead of the more commonly used >80 pg/mL as the Gothenburg microbiology department used that particular cut-off. However, there were only 15 BDG tests analyzed at the Gothenburg microbiology department and no samples analyzed at the Karolinska Department of Microbiology had BDG values ranging between 80 and 100 pg/mL. There is also an ongoing debate on whether higher cut-off levels may be preferable to increase the specificity of BDG [23]. Finally, the subcategorization of probable IAC based on receiving antifungal therapy must be interpreted with caution as both T2C and BDG results were made clinically available and could influence the choice to initiate antifungal therapy.

In conclusion, this study supports the use of T2C in the clinical management of severely ill surgical ICU patients at high risk of IAC. Due to a low sensitivity, T2C should preferably be used in combination with BDG. The T2C test is fast and has a high specificity, which is especially useful in critically ill patients where surgical interventions or antifungal adjustments are promptly needed.

## Figures and Tables

**Table 1:** Definition of proven IAC and probable IAC.

**Proven IAC**	Blood culture with the growth of *Candida* ssp. or Histopathologic, cytopathologic, or direct microscopic evidence consistent with *Candida* spp. in a specimen from a normally sterile site, obtained by needle biopsy or aspiration. or Culture with the growth of *Candida* spp. obtained by a sterile procedure, including <24 h drainage culture, from a normally sterile site showing a clinical or radiologic abnormality consistent with an infectious-disease process. and Clinical or radiologic abnormality consistent with an intraabdominal infectious process
**Probable IAC**	A patient having had gastrointestinal surgery or necrotizing pancreatitis admitted to the ICU or HDU and BDG ≥ 100 pg/mL

Abbreviations: IAC, intraabdominal candidiasis; BDG, 1,3-β-D-glucan; ICU, intensive care unit; HDU, high-dependency unit.

**Table 2:** Baseline characteristics, *n* = 134.

Male, *n* (%)	76 (57)
Age (years), median (IQR)	66 (57–73)
Renal failure, eGFR < 60 mL/min/1.73 m^2^ *	10 (7)
Immunosuppression, *n* (%)	27 (20)
Chemotherapy (67%)	
Solid-organ transplant (15%) High dose corticosteroids † (7%) Neutropenia (4%) Other (7%)	
Preceding gastrointestinal surgery, *n* (%)	116 (87)
Necrotizing pancreatitis, *n* (%)	18 (13)
*Multiple* abdominal *surgeries*, *n* (%)	26 (19)
30-day mortality, *n* (%)	19 (14)
Clinical data at first T2C/BDG testing	
SOFA score (average), ±SD	3.9 ± 3.5
Vasopressor treatment, *n* (%)	51 (38)
Invasive mechanical ventilation, *n* (%)	37 (28)
Admitted to the intensive care unit (%)	64 (48)
Admitted to the high-dependency unit (%)	70 (52)
Total parental nutrition, *n* (%)	39 (29)
Renal replacement therapy, *n* (%)	15 (11)
Broad spectrum antibiotic therapy, *n* (%)	78 (58)
Antifungal therapy, *n* (%)	17 (13)

Abbreviations: T2C, T2Candida magnetic resonance assay; BDG, 1,3-β-D-glucan; IQR, interquartile range; eGFR, estimated glomerular filtration rate; SOFA, sequential organ failure assessment; SD, standard deviation. * based on the MDRD formula (186.3 × (s-creatinine/88.4)^−1.154^ × age^−0.203^ (× 0.742 for female). † >20 mg prednisone equivalent a day.

**Table 3:** Performances of T2C and BDG for IAC criteria, *n* = 134.

	T2C+ (*n* = 10)	BDG+ (*n* = 31)
Proven IAC	6/13	11/13
IAC and candidemia	2/2	2/2
IAC no candidemia	4/11	9/11
Probable IAC	2/20	20/20 *
Negative IAC	2/101	0/101

Abbreviations: T2C, T2Candida magnetic resonance assay; BDG, 1,3-β-D-glucan; IAC, intraabdominal *candidiasis*. * Probable IAC was based on a positive BDG result.

**Table 4:** Diagnostic performances of T2C and BDG.

	Proven IAC *n* = 13		Proven + Probable IAC *n* = 33	Proven + Probable IAC (Treated *) *n* = 24	Proven + Probable IAC (Colonized †) *n* = 23
	T2C	BDG	T2C	T2C	T2C
Sensitivity	46% (19–75)	85% (55–98)	24% (11–42)	32% (15–54)	35% (17–57)
Specificity	97% (92–99)	83% (76–90)	98% (93–100)	98% (94–100)	98% (94–100)
**Positive Predictive Value**					
IAC prevalence 1%	12%	5%	11%	16%	16%
IAC prevalence 5%	42%	21%	39%	49%	50%
IAC prevalence 10%	61%	36%	58%	67%	68%
**Negative Predictive Value**					
IAC prevalence 1%	99%	100%	99%	99%	99%
IAC prevalence 5%	97%	99%	96%	97%	97%
IAC prevalence 10%	94%	98%	92%	93%	93%

Point estimates and 95% confidence intervals (in parentheses). Abbreviations: T2C, T2Candida magnetic resonance assay; BDG, 1,3-β-D-glucan; IAC, intraabdominal *candidiasis;* PPV, positive predictive value; NPV, negative predictive value. * Patients clinically deemed IAC and treated with antifungal therapy. † At least one supportive culture growing *Candida* spp. within ±7 days.

## Data Availability

The data presented in this study are available from the corresponding author upon request. The data are not publicly available due to ethical restrictions.
